# Fusiform versus Saccular Intracranial Aneurysms—Hemodynamic Evaluation of the Pre-Aneurysmal, Pathological, and Post-Interventional State

**DOI:** 10.3390/jcm13020551

**Published:** 2024-01-18

**Authors:** Jana Korte, Laurel M. M. Marsh, Sylvia Saalfeld, Daniel Behme, Alberto Aliseda, Philipp Berg

**Affiliations:** 1 Department of Fluid Dynamics and Technical Flows, University of Magdeburg, 39106 Magdeburg, Germany; 2 Research Campus STIMULATE, University of Magdeburg, 39106 Magdeburg, Germany; lmarsh23@gmu.edu (L.M.M.M.); sylvia.saalfeld@tu-ilmenau.de (S.S.); daniel.behme@med.ovgu.de (D.B.); philipp.berg@ovgu.de (P.B.); 3 Department of Mechanical Engineering, George Mason University, Fairfax, VA 22030, USA; 4 Department of Computer Science and Automation, Ilmenau University of Technology, 98693 Ilmenau, Germany; 5 University Hospital Magdeburg, University of Magdeburg, 39106 Magdeburg, Germany; 6 Department of Mechanical Engineering, University of Washington, Seattle, WA 98195, USA; aaliseda@uw.edu; 7 Department of Medical Engineering, University of Magdeburg, 39106 Magdeburg, Germany

**Keywords:** computational fluid mechanics, flow diverting stents, fusiform intracranial aneurysm, hemodynamics, virtual stenting, saccular intracranial aneurysm

## Abstract

Minimally-invasive therapies are well-established treatment methods for saccular intracranial aneurysms (SIAs). Knowledge concerning fusiform IAs (FIAs) is low, due to their wide and alternating lumen and their infrequent occurrence. However, FIAs carry risks like ischemia and thus require further in-depth investigation. Six patient-specific IAs, comprising three position-identical FIAs and SIAs, with the FIAs showing a non-typical FIA shape, were compared, respectively. For each model, a healthy counterpart and a treated version with a flow diverting stent were created. Eighteen time-dependent simulations were performed to analyze morphological and hemodynamic parameters focusing on the treatment effect (TE). The stent expansion is higher for FIAs than SIAs. For FIAs, the reduction in vorticity is higher ( Δ 35–75% case 2/3) and the reduction in the oscillatory velocity index is lower ( Δ 15–68% case 2/3). Velocity is reduced equally for FIAs and SIAs with a TE of 37–60% in FIAs and of 41–72% in SIAs. Time-averaged wall shear stress (TAWSS) is less reduced within FIAs than SIAs ( Δ 30–105%). Within this study, the positive TE of FDS deployed in FIAs is shown and a similarity in parameters found due to the non-typical FIA shape. Despite the higher stent expansion, velocity and vorticity are equally reduced compared to identically located SIAs.

## 1. Introduction

Intracranial aneurysms (IAs) are a pathological dilatation occurring in the neurovascular arteries. These IAs carry the risk of rupture, which can lead to subarachnoid hemorrhages resulting in high rates of morbidity or even mortality [1]. Many IAs can be classified as saccular IAs (SIA) due to their balloon-like shape with an identifiable neck separating the aneurysm from the host parent vessel. Hemodynamics and treatment effects within SIAs are part of many past and current studies [2,3,4]. Furthermore, the treatment procedure is well studied and applied in neurointerventional surgery [5,6,7,8,9]. A non-saccular aneurysm, where the aneurysm typically affects the entire circumference of the vessel wall, is referred to as a fusiform IA (FIA). FIAs carry, in addition to rupture risk, the risk of ischemia, due to internal branch occlusion or distal embolization [10,11,12]. Therefore, when treating FIAs, the focus lies on restoring the arterial lumen, on preserving the flow inside the FIA and its internal arteries, and on the prevention of occluding vessels internally branching off within the IA [13]. According to al-Yamany et al. and Park et al. [14,15], FIAs occur within all IAs with an incidence of 3%. Due to this low incidence rate, most studies focus on the typical shape of FIAs when investigating intra-aneurysmal flow and treatment effect [16,17]. The treatment effect on hemodynamic parameters within FIAs that do not show conventional FIA shape need further investigation.

Endovascular therapy, such as the deployment of a flow-diverting stent (FDS), reduces the rupture risk of SIAs by effecting the blood flow entering the aneurysm [8,18]. The goal is to bring the intra-aneurysmal flow to stasis by allowing a thrombus formation to occlude the aneurysm. The wall shear stress (WSS) along the scaffolding is restored to that of the original parent vessel prior to the pathological growth [19], while the goal of FIA treatment is identical, there are few studies to confirm that the hemodynamic outcomes are the same between FIAs and SIAs when treated with a FDS [20,21,22,23]. As found by Barletta et al., flow diversion seems to show most promising results concerning the treatment of FIAs [21]. Saalfeld et al. analyzed FIA’s hemodynamics qualitatively within pathological, healthy, and treated cases [20]. Lv et al. investigated FIA morphology and stent deformation when applying virtual multiple treatment [24]. Griffin et al. performed a clinical study analyzing the outcome of FIA treatment with flow diverter stents focusing on the occlusion rate captured with varying clinical imaging methods [25]. They also compared the occlusion to the treatment outcome of FDS deployed in SIAs and found higher rates in the latter. However, the focus on hemodynamic parameters in FIAs compared to SIA flow and the treatment impact on these parameters is missing in these studies.

Image-based blood flow simulations using computational fluid dynamics (CFD) and virtual treatment approaches provide a great opportunity to model the hemodynamics and the treatment effect without requiring any extra procedure or inconvenience to the patient [26,27,28].

To enhance the knowledge of hemodynamics in non-typically shaped FIAs, this study focused on analyzing these in comparison to equally located SIAs. Thus, the locations within the Circle of Willis do not play a role within the comparison between FIAs and SIAs. Six patient-specific IAs are taken into account in this study. Besides the pathological hemodynamics, the treatment effect and the physiological flow was analyzed and compared among the FIA and SIA cases. To study this, each case was virtually treated with a FDS and the healthy counterpart manually created. The aim is to understand the difference in the flow fields within non-typically shaped FIAs compared to SIAs and the comparability of the impact of treatment onto these flow fields, respectively.

## 2. Materials and Methods

### 2.1. Patient Cohort

**Figure 1:**
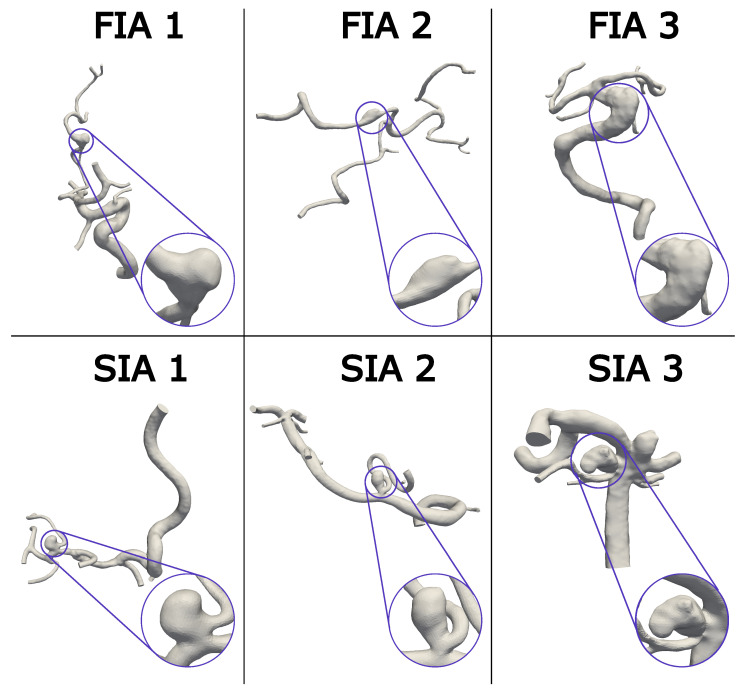
Visualization of the aneurysm cohort with the FIAs in the first row and SIAs in the second row. A magnification of each IA is provided, to give an overview of the different shapes. Notice the complex vasculature considering the patient-specific anatomy proximal and distal to the IAs.

Six patients in total were enrolled in this study, including three FIA and SIA cases occurring in the Circle of Willis, respectively, (FIA/SIA 1–3). The chosen FIA and SIA cases 1–3 are shown in Figure 1 with the FIAs in the top and the SIAs in the bottom, comprising different shapes and sizes. The whole vasculature is shown with a detailed IA view for each case. Regardless of the aspect ratio, SIAs typically exhibit a small neck when compared to the volume of the dome or another length scale of the aneurysm. However, FIAs are, by definition, circumferential, which means the neck area to aneurysm volume ratio is larger than for SIAs and the vessel is affected all around [14,29]. This means the flow has an increased chance of gently expanding into an untreated fusiform aneurysm rather than a saccular case in which an inflow jet creating a wall-impingement zone is not uncommon. Because FIAs are so rare, only three could be obtained with appropriate imaging from a database of at least 300 IAs. Nevertheless, due to a non-balloon-shape without an entire circumferential extension of the FIA vessel, these FIAs feature a non-typical shape.

From the mentioned available database, the choice of the opposed SIA was made based on similar artery (Middle Cerebral Artery—MCA, Vertebral Artery—VA, Basilar Artery—BA), location on and diameter of the vessel (to match flow rates), and size (aspect ratio (AR) and volume) in that order. Although we were searching the database of 300 IAs, not every criterion to match the location could be met exactly and a few drawbacks such as the location on different branches had to be accepted. Even with this database, not all cases were captured with the same imaging technique. The corresponding SIA was chosen to match the FIA as closely as possible. FIA/SIA 1 are located at the MCA and were captured with three-dimensional digital subtraction angiography (3D DSA) with a resolution of 0.28 × 0.28 × 0.28 mm^3^. Case 2 is located at the left VA. FIA 2 was captured with computer tomography with a resolution of 0.47 × 0.47 × 0.47 mm^3^ and SIA 2 with 3D DSA at a resolution of 0.15 × 0.15 × 0.15 mm^3^. Case 3 is located on the BA, and FIA 3 was captured with 3D DSA (0.36 × 0.36 × 0.36 mm^3^). The corresponding SIA 3 was imaged using 3D DSA as well with a resolution of 0.28 × 0.28 × 0.28 mm^3^.

### 2.2. Virtual Stenting, Vessel Reconstruction, and Aneurysm Sac Definition

To allow for an overall population comparison, as well as a comparison within each case, the hemodynamics in each IA are compared to the hemodynamics of the same patient within the treated vasculature and the healthy vasculature (aneurysm virtually removed from the parent vessel).

**Figure 2:**
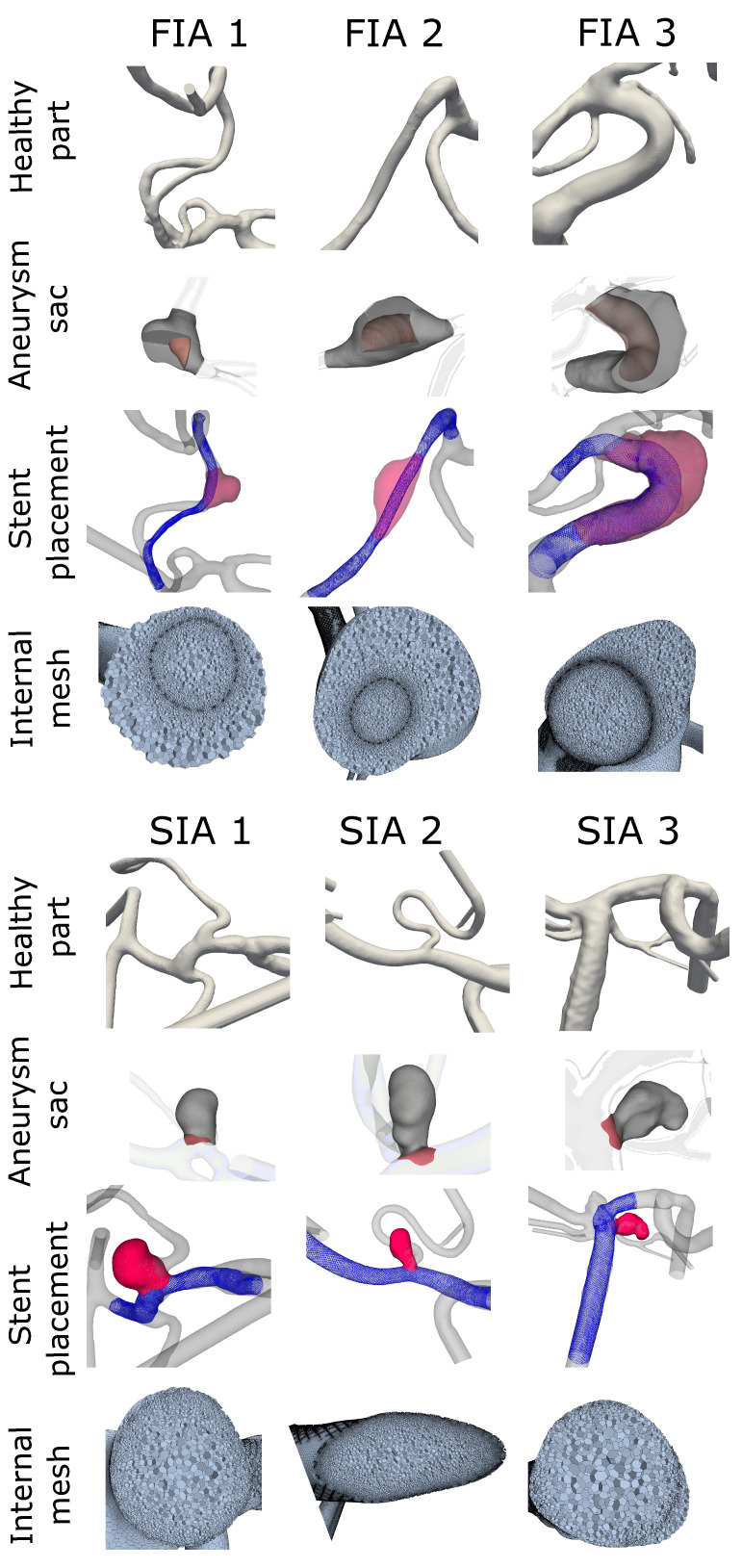
Aneurysm cohort depicting FIAs in the top four rows and SIAs in the lower four rows with the manually created healthy vasculature (healthy part) on top rows. Second rows show resulting aneurysm domain, used for post processing (aneurysm sac). The sac is shown in gray with surfaces within the FIA cases to visualize the internal boundary of the vessel. The treated version of FIA/SIA is shown in the third row with the pathological aneurysm sac in red and the FDS in blue (stent placement). In the last row an insight into the internal mesh used for the hemodynamic simulation is presented.

A previously-verified fast virtual stenting approach [30] was applied allowing different stent configurations to be replicated in each case within minutes on a personal laptop. The software is based on geometric deformations and produces realistic deployment with decent wall apposition. First, the geometry was discretized and voxelized before a centerline was created and optimized. The stent was then deployed without retaining the circular cross-section which allows for the shortening or lengthening based on the average radius along the deploying stent. A PED, comprised of 48 wires with a tine radius of 19 μ m, was modeled for each case. This practice allowed for precise placement and positioning within the vessel that will affect the flow in a realistic manner (see Figure 2, FDS shown in blue). Stent sizing for each case was determined by an experienced neurointerventionalist.

To restore the original parent vessel, prior to aneurysm initiation and growth, a healthy version of each case was created with the guidance of the same neurointerventionalist. For this recreation, the aneurysm sac was manually removed from the vessel using the open-source design software Blender (v2.82, Blender Foundation, The Netherlands). The remaining vessel was then smoothed to match the parent vessel surface. Moreover, under consideration of the complete vasculature the healthy vessel formation was manually reconstructed (see Figure 2: healthy part). Since this was carried out without using automated tools, the precision was checked and achieved by consulting a neurointerventionalist.

To perform a precise analysis of the flow-related parameters, the volumetric aneurysm sac and the accompanying dome area were defined for each case. The healthy vessel aided in the definition of the aneurysm sac. First, the healthy vessel was expanded equally in all directions until it was fully intersecting the aneurysm, occluding it from the parent vessel. The healthy vessel volume was then cut from the pathological geometry, so that only the aneurysm sac (volume and surface) remained. Second, if any bifurcating arteries were coming from the aneurysm, they were virtually removed and smoothed. The remaining domain, which was then defined as the aneurysm for post-processing purposes, is presented in Figure 2 (aneurysm sac).

### 2.3. Hemodynamic Simulation

Each case, FIA/SIA 1–3, was analyzed for the pathological (P), treated (T), and healthy (H) condition. Overall, 18 hemodynamic simulations were carried out within this study to analyze the intra-aneurysmal flow within FIA/SIA. For the spatial discretization as well as for the simulations the fluid dynamics solver StarCCM+ (v2021.1, Siemens, Munich, Germany) was used. The mesh has a global minimum and base size of 0.0375/0.15, 0.0375/0.15, and 0.01/0.2 mm for the H, P, and T models. Tetrahedral cells along with 3 boundary layers expanding at a rate of 1.3 were used to construct the H and P models. The stent was meshed to a precision between 0.01 and 0.02 mm, then polyhedral cells with a stretch of 1.3 were used to mesh the T models. This has been shown to be sufficient by a previous mesh convergence study of treated and untreated models [31]. The resulting cell count ranged from 0.27 to 2.94 million cells for the untreated cases and from 0.44 to 9.03 million cells for the treated ones.

Walls were assumed to be rigid and blood to be incompressible and Newtonian with a density of 1055 kg/m^3^ and a viscosity of 0.004 Pa·s .

Inlet boundary conditions were set transient with a mass flow rate captured from a healthy proband in a previous study [32]. For each model, the same mass flow rate was used, with the magnitude scaled according to the specific inlet cross sectional area [33]. This is to prevent the same impact of the mass flow rate onto the inlet flow velocity. Inlets were extended to ensure a fully developed flow profile [34]. A splitting value was set at each outlet calculated with an in-house flow splitting method specific for each vessel model [35]. The splitting method was previously proofed to be stable and realistic [36].

### 2.4. Morphological and Stent Analysis

Stent and vessel analysis were performed using VMTK (v1.3, vmtk.org) to obtain centerlines and radii. StarCCM+ and MATLAB (R2022a, MathWorks Inc., Natick, MA, USA) were used to create envelopes of the stent, aneurysm, and vessel volumes. Matlab was also used to process the data. Regarding the morphological analysis, the aneurysm volume and stent area were calculated using the assessed envelopes. From this, the area to volume ratio was derived. Concerning the stent analysis, the mean and maximum stent expansion was derived for each case. This stent expansion was calculated based on the deployed stent diameter compared to the given stent diameter using a percent change score

(1) $$\begin{array}{c} Stent\;expansion=\left(\frac{Deployed\;stent\;diameter}{Nominal\;stent\;diameter}\right)\times 100. \end{array}$$

The nominal diameter was the manufacturer’s specification for the optimal vessel size the stent should be deployed in. For the analysis, the stent expansion was only considered along the part of the vessel comprising the aneurysm neck. This is because stent lengthening or foreshortening can be drastically changed based solely on the vessel morphology. This allows for a direct comparison between the morphological response of SIAs and FIAs to the treatment.

### 2.5. Hemodynamic Analysis

Parameter values were qualitatively and quantitatively evaluated in EnSight (2019 R3, ANSYS Inc., Canonsburg, PA, USA) and in MATLAB. The metrics were separated into aneurysm dome wall parameters and flow-related volumetric parameters. Raw values were compared as well as the treatment effect (*TE*) which is the percent change for a given metric *x*

(2) $$\begin{array}{c} TE=\frac{x_{post}-x_{pre}}{x_{pre}}\times 100. \end{array}$$

Mean values describe the spatial average within the aneurysm part. All flow-related parameters were calculated only within the aneurysm sac for FIA and SIA, as described in Section 2.2.

Concerning the wall parameters, time-averaged wall shear stress (*TAWSS*) and oscillatory shear index (*OSI*) were considered. Due to their proven relevance, these were commonly studied in IA rupture analysis [37,38].

The *TAWSS* is the wall shear stress averaged over one cardiac cycle $T_{c}$ . Cycle length $T_{c}$ depended on the inflow rate (see Section 2.3) 

(3) $$\begin{array}{c} TAWSS=\frac{1}{T_{c}}\int_{0}^{T_{c}}WSS\;dt. \end{array}$$

*OSI*, the fluctuation of *WSS* over the cardiac cycle, was determined by the change in orientation and magnitude of *WSS*

(4) $$\begin{array}{c} OSI=0.5\times \left(1-\frac{\left|\int_{0}^{T}WSS\;dt\right|}{\int_{0}^{T}\left|WSS\right|\;dt}\right). \end{array}$$

OSI ranges from 0 to 0.5 where an increasing value of *OSI* indicates a high level of fluctuation. The volumetric flow-related parameters of interest were energy loss (*EL*), time-averaged velocity (*v*), time-averaged vorticity ( ω ), and oscillatory velocity index (*OVI*); these allow the analysis of intra-aneurysmal blood flow [4,37,39]. *v* and ω were averaged over one cardiac cycle.

*OVI* indicates the fluctuation of the velocity vector over one cardiac cycle. Similar to *OSI* on the wall, the calculation of *OVI* within the dome was based on the change in orientation of the velocity vectors and their magnitudes

(5) $$\begin{array}{c} OVI=0.5\times \left(1-\frac{\left|\int_{0}^{T}v\;dt\right|}{\int_{0}^{T}\left|v\right|\;dt}\right). \end{array}$$

The effects of the aneurysm formation on the blood flow were analyzed using the *EL*. Based on *v*, surface area (*A*), fluid density ( ρ ), and static pressure (*p*), the energy (*E*) at the inlets and outlets was calculated for each case

(6) $$\begin{array}{c} E=Av\left(\frac{1}{2}\rho v^{2}+p\right). \end{array}$$

The *EL* was estimated as the difference between *E* on a plane proximal to and distal to the aneurysm within each vessel model. Thus, the sum of the proximal *E* was subtracted from the distal *E* [40]

(7) $$\begin{array}{c} EL=\sum \;E_{inlets}-\sum \;E_{outlets}. \end{array}$$

The resulting *EL* presents the difference between pathological (*P*), treated (*T*), and healthy (*H*) vessel models.

## 3. Results

### 3.1. Morphological and Stent-Related Differences

In Table 1, (column 1–3) the aneurysm volume, the stent area, and their ratio are shown for each case comparing FIA and SIA, respectively. Aneurysm volume was higher for FIA 2/3, but lower for FIA 1. Nevertheless, the stent area was consistently higher for the FIA in each case; this is also true for the area/volume-ratio.

Due to the manufacture’s specification of the optimal vessel size, the stent’s conditions were chosen by the neurosurgeon based on the IA location and associated vessel size. The values of the virtual expansions are summarized in Table 1 (column 4–6) along with the nominal diameter. Both the mean and maximum expansion of the FDS while crossing the IA feature a higher expansion of the stent within FIAs than SIAs. Especially the stent for FIA 2 expanded past the given diameter of 3 mm to 3.64 mm while crossing the IA, which results in an expansion of 122%. The analogue SIA 2’s 4.5 mm stent expands to the given diameter of 4.64 mm which was considered a 103% expansion.

### 3.2. Hemodynamic Differences

**Figure 3:**
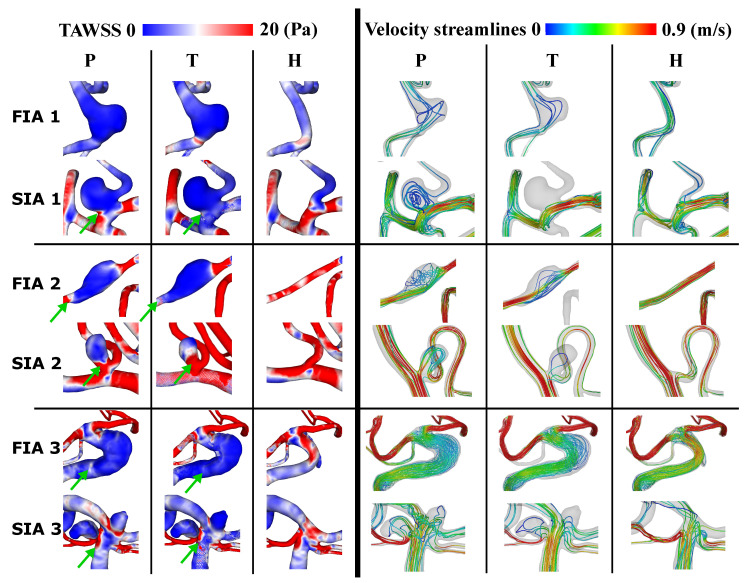
Resulting TAWSS on aneurysm surface (**left**) and streamlines showing average velocity within vessel models (**right**) for cases 1–3 for pathological (P), treated (T) and healthy (H). Lower TAWSS at IA neck and border within T than P is marked with a green arrow.

Figure 3 shows the resulting TAWSS on the vessel surfaces for the three FIA and SIA cases and each condition (P, T, H) on the left and the according streamlines colored with the mean v on the right. The TAWSS appears lower on the aneurysm dome after treatment for FIA and SIA compared to the pathological part. This applies especially for the aneurysms’ necks and borders (marked with green arrows in Figure 3). The healthy counterparts comprise higher TAWSS for both FIA and SIA when compared to T and P. Flow diversion is clearly visible from P to T when looking at the streamlines. Especially within the FIAs, from P to T streamlines are straightened and v seems higher. When considering H compared to P and T, v is highest. Within SIA 2/3 the streamlines still pass the aneurysm when comparing P to T and v is not noticeably increased. Within H of the SIA 2/3, v is higher than within P and T, as was also observed for the FIAs.

**Figure 4:**
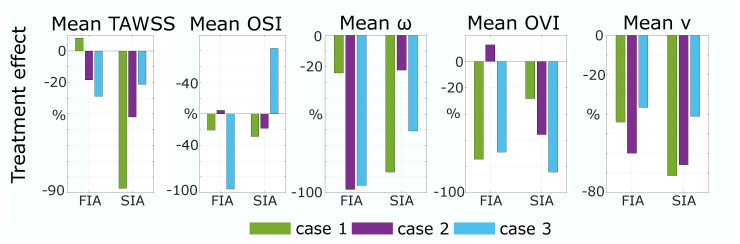
Resulting Treatment Effect: The change in metrics for cases 1–3 from pathological to treated of TAWSS and OSI on the aneurysm surface and of mean ω , mean OVI, and mean velocity (v) inside aneurysm sac.

The quantitative results strengthen the assumption drawn from Figure 3. As shown in Figure 4 (left), the reduction in TAWSS from P to T is higher than 19% for FIA 1 and 2. For SIAs, the TE of TAWSS is higher than 20% for all cases and, compared to FIAs, SIA 1 and 2 present higher values than FIA 1 and 2. OSI is crucially reduced by treatment of FIA 1/3 (FIA 1: 20%, FIA 3: 95%) and slightly increased within FIA 2 (5%). Concerning SIAs, OSI is reduced for SIA 1/2 (SIA 1: 20%, SIA 2: 30%); a crucial increase of 80% appears within SIA 3. For the quantitative results of the mean values of v, ω and OVI the temporally and spatially averaged metrics are shown for each case 1–3 in Figure 4 on the right. The ω was reduced by treatment for all cases and an equal impact was presented for SIA and FIA treatment. Still, results for FIA 2/3 show a higher mean ω reduction (FIA 2: 95%, FIA 3: 97%) with treatment than for SIA 2/3 (SIA 2: 22%, SIA 3: 61%). Concerning OVI, a reduction was detectable for all cases (≥27%) despite FIA 2 (+13%). With treatment the mean v is reduced drastically and similar for SIAs and FIAs, being slightly higher ( Δ 5–30%) for SIAs.

The resulting EL for each case and condition is shown in Table 2. Looking at the pathological cases, EL is lowest for case 1, higher for case 2, and highest for case 3. This applies for FIA and SIA. Within FIA 1 and FIA 2 the EL is higher for the healthy than for the pathological cases; this is not the case for FIA 3, where EL is lower for the healthy case. Also, within SIA 1–3 EL is lower within the healthy cases than within the pathological ones. Concerning the treated IAs, EL is higher than healthy and pathological for all SIAs and for FIA 2. For FIA 1/3, EL is lowest within the treated version.

## 4. Discussion

While the investigation of IAs increased in recent years, especially due to improving medical imaging technologies and numerical simulations, the focus mostly lies on saccular shapes. Deploying FDS as a minimally-invasive endovascular treatment of SIAs was established and the reduction of the flow into the SIA has been presented in many studies [6,41,42]. Fusiform-shaped IAs in the cerebrovascular circulation are as of yet not well studied, and knowledge about the flow field and treatment effect is still low [20]. The aim of this study was to analyze FIAs in comparison to SIAs focusing on the treatment with FDS.

### 4.1. Morphology and Stent

The virtual stent analysis shows an increased deployed diameter past the given diameter for all FIAs. The mean stent expansions follow a similar trend for all cases with stronger values across the entire FIA than the corresponding saccular ones. The ability of the stent to freely expand circumferentially in the FIAs lends itself to this greater max and mean expansion. Since the stent area and length for FIAs are substantially larger (see Table 1) one outcome of over-expansion, even just within the IA, is foreshortening. Another, even greater concern is that the porosity increased and allowed a higher flow into the IA. This is visible in the qualitative and quantitative hemodynamic results (recall Figure 3 and Figure 4, which show, in general, a lower TE for FIA than SIA. Rhee et al. considered, that even though with decreasing porosity the intra-aneurysmal flow was positively affected; however, this effect was not significant [43]. Compared to typically shaped FIAs, the stent expansion was lower [21].

### 4.2. Wall-Related Parameters

As Rhee et al. found, FDS have a higher impact on WSS reduction within SIAs than FIAs [43]; within this study, a higher impact onto TAWSS within SIAs than FIAs was found as well. Nevertheless, the non-typical shape of the investigated FIAs lead to a better TE in general when comparing to the literature [43]. FIA 1 has an increase of WSS which appears to be a direct result of the flow diverted away from the treated vessel branch, and less from the aneurysmal apex itself. Moreover, FIA 1 has featured the highest stent expansion. However, because of the nature of FIAs, this change in TAWSS to the treated vessel/aneurysmal sac can be important to the FIA growth [44]. FIA 2 experiences an increase in OSI. This post-treatment increase in oscillation appears in a low-velocity region and also seems to coincide with a high-OSI surface in the treated case. This can be related to the findings of Takehara et al. [45], who found higher OSI in regions with low velocity and decreased WSS when analyzing aortic hemodynamics with four-dimensional flow magnetic resonance imaging. SIA 3 also showed a strong increase in OSI which occurs at the apex of the IA dome where the already low WSS may be changing directions based on minor deviations in the flow throughout the cardiac cycle (recall the streamlines in Figure 3) [23]. OSI showing variable increase or decrease when applying FDS in FIAs agrees with Lv et al. [24].

### 4.3. Flow-Related Parameters

Concerning the hemodynamic results, treated IAs generally see equal reductions of the velocity for FIAs and SIAs (recall Figure 3 and Figure 4). Concerning SIA reduction, this was already presented in the literature [2]. The streamlines illustrate that the flow is redirected through the parent vessel part by FDS in SIAs, which is in accordance with Sindeev et al. [23]. This might be affected by the non-typical shape of the non-typical circumferential FIAs as well. This shows that the circumferential shape has to be taken into account additionally when defining the treatment method based on the aneurysmal geometry. The overall higher reduction in ω within FIAs, leads to the assumption, that with FDS, flow structures were straightened as well, which can also be derived from Figure 3. Lv et al. [24], who investigated multiple treatment in FIAs, stated that with FDS the vortex formation inside the IA shifts from the wall to the vessel center, which agrees with the findings in this study: OVI and ω are decreased with treatment, leading to the fact that vortex formation is decreased as well. Interestingly, FIA 2 has one of the largest reductions in ω , but shows an increase in OVI. Again, this might be related to the reduced velocity, since fluctuations of lower velocities within the IA lead to an increase in OVI. Furthermore, this could be due to the non-typical shape of the FIA as mentioned regarding the wall parameters.

### 4.4. Energy Loss

Concerning the pathological to healthy comparison of the EL, lower EL occurs within FIA and higher EL within SIA compared to the healthy counterpart. In this study, the treatment increases the EL drastically for all SIAs and decreases the EL in FIA 1/3. Chong et al. found out that reduced EL has been correlated with favorable outcome of stent-treated endovascular therapies [46]. This indicates that due to treatment, the flow energy is converted into fluctuations within the underlying cases. Looking at FIA 1/3, the reduction indicates a favorable outcome of the treatment. However, for FIA 2 the treatment increases the EL. The lower EL within untreated pathological FIAs, is an interesting, but doubtful outcome. Since compared to a previous study, the resulting EL values for SIAs are higher for FIA/SIA 2/3 (≈50%) [40], the EL results may not give crucial hints on successful treatment effect for the underlying cases.

### 4.5. Limitations and Future Work

First, due to a lack of information, the numerical simulations underlying assumptions (no patient-specific inflow curves, blood assumed Newtonian, rigid walls) and IA treatment could not be visualized in vivo. Nevertheless, within the literature, validation of numerical simulations is performed in several studies and the use of CFD is well-established in patient-specific blood flow analysis [32,37]. Second, only a small number of cases was analyzed. Since FIAs occur only with a prevalence of approx. 3% of all IAs [15] and are rarely captured by medical imaging so far, this is nevertheless a progress in their investigation [20,47]. Third, regarding the virtual stenting and vessel reconstruction only one FDS version was virtually placed into the IAs neglecting the analysis of differences in porosity and no proof of the healthy vessel was available. This could help in finding reasons for less TAWSS reduction and possibilities for higher overall flow reduction. Fourth, the deployment of FDS in SIAs is not the best possible treatment and only virtually applied in this study to preserve the comparable basis of the treatment outcome. Future work will therefore include the comparison of different treatment option in SIAs and FIAs. Moreover, variation in stent porosity and a multiple stent analysis to extend the knowledge about the impact of FDS onto FIAs will be considered. Moreover, to strengthen these findings, a higher number of cases should be analyzed; in vitro as well as in vivo validation is required. Despite these limitations, the compatibility of FDS in non-typically shaped FIAs is presented within this study. The comparable reduction in velocity from FDS within FIAs and SIAs is shown.

## 5. Conclusions

FDS within FIAs show higher expansion and therefore a possible reduction in porosity when compared to SIA implantation. Nevertheless, FDS affect FIAs and SIAs equally when considering flow reduction, namely velocity and vorticity. Still, TAWSS and OVI reduction is higher for SIAs. FIAs with a non-typical circularly shape show a treatment outcome closer to the SIA treatment outcome for specific parameters, which should be taken into account when deciding on the treatment method of an FIA. Still, no significant statement could be derived from the low case count, which will be increased in future work to reach a significant outcome. Moreover, the analysis could be extended by adding multiple (double or triple) FDS to find out if this leads to stronger TAWSS reduction in FIAs.

## Figures and Tables

**Table 1:** Morphological parameters of aneurysm volume and exposed stent area and stent expansion to illustrate the differences between SIA and FIA.

Case	Aneurysm Volume (mm^3^)	Stent Area (mm^2^)	Area/ Volume (1/mm)	Nominal Stent Diameter (mm)	Max Stent Expansion (%)	Mean Stent Expansion (%)
FIA 1	30.4	38.6	1.3	2.3	104	91
SIA 1	33.2	7.84	0.2	2.3	91	82
FIA 2	159	168	1.1	3.0	122	87
SIA 2	52.7	6.35	0.1	4.5	103	58
FIA 3	920	656	0.7	7.0	103	80
SIA 3	34.1	4.4	0.1	4.0	83	72

**Table 2:** Resulting Energy Loss for each case and condition in Watt (W).

Case	EL (W)		
	P	T	H
FIA 1	−8.17 $\times \;10^{-6}$	−9.57 $\times \;10^{-6}$	4.60 $\times \;10^{-5}$
SIA 1	5.02 $\times \;10^{-5}$	8.16 $\times \;10^{-5}$	3.79 $\times \;10^{-5}$
FIA 2	2.69 $\times \;10^{-3}$	4.96 $\times \;10^{-3}$	3.22 $\times \;10^{-3}$
SIA 2	1.79 $\times \;10^{-3}$	2.65 $\times \;10^{-3}$	1.76 $\times \;10^{-3}$
FIA 3	9.50 $\times \;10^{-3}$	−5.17 $\times \;10^{-3}$	9.33 $\times \;10^{-3}$
SIA 3	2.48 $\times \;10^{-3}$	2.91 $\times \;10^{-3}$	1.64 $\times \;10^{-3}$

## Data Availability

Data are available under reasonable request.

## Abbreviations

The following abbreviations are used in this manuscript:

A	Surface Area
AR	Aspect Ratio
BA	Basilar Artery
CFD	Computational Fluid Dynamics
DSA	Digital Subtraction Angiography
E	Energy
EL	Energy Loss
FDS	Flow Diverting Stent
FIA	Fusiform Intracranial Aneurysm
H	Healthy
IA	Intracranial Aneurysm
MCA	Middle Cerebral Artery
VA	Vertebral Artery
OVI	Oscillatory Velocity Index
OSI	Oscillatory Shear Index
P	Pathological
p	Pressure
SIA	Saccular Intracranial Aneurysm
T	Treated
TAWSS	Time Averaged Wall Shear Stress
$T_{c}$	Cardiac Cycle Length
v	velocity
W	Watt
WSS	Wall Shear Stress
ρ	Fluid Density
ω	Vorticity
